# Origin and classification of spontaneous discharges in mouse superficial dorsal horn neurons

**DOI:** 10.1038/s41598-018-27993-y

**Published:** 2018-06-27

**Authors:** Javier Lucas-Romero, Ivan Rivera-Arconada, Carolina Roza, Jose A. Lopez-Garcia

**Affiliations:** Department of Systems Biology, Universidad de Alcala, Alcala de Henares, 28871 Madrid, Spain

## Abstract

Superficial laminae of the spinal cord possess a considerable number of neurons with spontaneous activity as reported *in vivo* and *in vitro* preparations of several species. Such neurons may play a role in the development of the nociceptive system and/or in the spinal coding of somatosensory signals. We have used electrophysiological techniques in a horizontal spinal cord slice preparation from adult mice to investigate how this activity is generated and what are the main patterns of activity that can be found. The results show the existence of neurons that fire regularly and irregularly. Within each of these main types, it was possible to distinguish patterns of spontaneous activity formed by single action potentials and different types of bursts according to intra-burst firing frequency. Activity in neurons with irregular patterns was blocked by a mixture of antagonists of the main neurotransmitter receptors present in the cord. Approximately 82% of neurons with a regular firing pattern were insensitive to synaptic antagonists but their activity was inhibited by specific ion channel blockers. It is suggested that these neurons generate endogenous activity due to the functional expression of hyperpolarisation-activated and persistent sodium currents driving the activity of irregular neurons.

## Introduction

Spontaneous activity of superficial dorsal horn neurons has been reported in acute preparations using monkeys^1^, cats^2^ and rats^3^ as well as in several *in vitro* preparations^4,5^. Some authors have considered spontaneous activity as random noise that should be subtracted from the actual evoked responses^6^. However, a few reports have focused on the study of such activity. The general picture emerging from these studies suggests that spontaneous activity is not random (not always, at least) and that it may play an important role in the coding of sensory information.

Using an acute cat preparation, Cervero *et al*.^7^ reported the existence of spinal LII neurons with persistent but irregular spontaneous activity in the 5–10 Hz frequency range. These neurons showed ‘inverted’ responses to innocuous and/or nociceptive mechanical stimulation of the skin consisting on the inhibition of ongoing activity. The authors proposed that they could exert a tonic inhibition on projecting neurons and therefore their inhibition would enable or facilitate the excitation of these projecting neurons. Using the acute rat preparation, Sandkühler & Eblen-Zajjur^3^ reported the existence of rhythmically and non-rhythmically firing neurons in superficial and deep laminae. Rhythmic neurons fired at low frequencies with a bimodal distribution (2 Hz or 10 Hz) and some of them fired bursts rather than single spikes. These neurons were typically local network interneurons and their activity was supposed to be generated and maintained by synaptic activity within the networks. Although the anaesthetics used did not appear to modify the percentage of rhythmic neurons, rhythmic firing could be modulated by substance P and spinalisation^8,9^. Authors speculated with the possibility that this rhythmic activity could serve to the purpose of information processing within the cord and/or to provide trophic signals to postsynaptic neurons.

Li & Baccei^4^ distinguished irregular, tonic and bursting neurons in LI of rat spinal cord slice preparations. Both tonic and bursting neurons were defined as rhythmic by visual inspection of recordings. Bursting behaviour was supposed to be exclusive of LI interneurons which send their axons to deeper layers, including the ventral horn, and use glutamate as neurotransmitter. However, larger neurons with this pattern were found to project to brainstem nuclei^10^. An important contribution of this work was to show that burst firing persisted in the presence of antagonists of fast excitatory and inhibitory transmitters (see also Luz *et al*.^11^). This suggested an intrinsic mechanism of oscillation in which persistent sodium currents as well as voltage gated calcium channels, calcium activated potassium currents and inward-rectifying potassium currents could be involved^4,12^. Bursting neurons received monosynaptic input from high threshold fibres and responded to electrical stimulation with large bursts when stimulated at inter-burst periods but did not show clear responses when stimulated within the burst period. Authors proposed that the function of these neurons may be to supply synaptic input to neonatal sensory-motor circuits to facilitate its development, since they are more frequently found at P2–3 than at P20–23.

Recently we have reported the existence of several patterns of spontaneous activity in superficial dorsal horn of mice using spinal cord slice preparations and a restrictive statistical tool to establish regularity^5^. These included several irregular patterns (single spike, slow burst, fast burst and mixed burst) as well as one regular pattern which we termed clock-like and may be equivalent to the tonic type of Li & Baccei^4^ and some of the rhythmic neurons of Sandkühler & Eblen-Zajjur^3^. The number of clock-like neurons and the probability of synchronic firing was increased in animals that had undergone nerve injury^5^, indicating that neurons with spontaneous activity may be involved in the abnormal coding of sensory information that may take place under sensitised conditions.

All these previous reports on the spontaneous activity of spinal neurons tend to focus attention on a single specific pattern, ignoring or just mentioning other patterns. Additionally, a full description of patterns or even a mathematical analysis of regularity is not always available, contributing towards a lack of clarity in the field. This is in contrast with the increasing interest that spike train analysis is gaining to understand better the neural coding of sensory information^13^. In view of the dispersion of data and the different names and traits attributed to superficial dorsal horn neurons, here we set out to create a new systematic nomenclature for neurons according to the pattern of spontaneous activity shown in the absence of stimulation. We also aimed at establishing the role of synaptic and intrinsic factors in the generation of spontaneous activity for each type of neuron and to evaluate the effects of simple strategies of selective blockade on the spinal processing of afferent inputs.

In the present report we have used arrays of electrodes to record from several neurons at a time, combined with a rigorous but flexible analysis of the regularity of firing with the coefficient of variation of inter spike intervals. We have also used whole cell recordings to validate specific ion channel blockers under our experimental conditions. The results obtained allow the proposition of a comprehensive nomenclature that identifies with clarity each pattern of activity and establish a role for persistent sodium (I_NaP_) and hyperpolarisation-activated (I_h_) currents in the organisation of intrinsic rhythmicity underlying the regularity of firing in a proportion of neurons which in turn may drive other non-regular neurons.

## Results

### Characterisation of the sample of neurons tested

The present studies include a total of 371 neurons recorded with MEAs. According to data on the depth of electrode tracking, we estimate that our recording sites were placed in the superficial laminae I-III of the dorsal horn. We did not observe any systematic relation between the type of neuron recorded with depth or placement of electrodes. Electrodes with 16 recording points yielded 3–5 simultaneous recordings from well isolated neurons. The yield of 32 recording site electrodes was 10–12 neurons.

An additional group of 150 neurons were recorded with patch pipettes in whole cell configuration using identical experimental settings and recording locations.

### Patterns of spontaneous activity found in dorsal horn neurons with MEAs

Details about the neuronal classification proposed, numbers of neurons per class, firing frequency and coefficient of variation (CV) are shown in Table 1.

**Table 1:** Distribution of neurons per types of firing patterns.

	IS	IFB	ISB	IMB
No. Neurons	185	74	25	12
% total	49.9%	19.9%	6.7%	3.2%
Mean frequency in 5 min (Hz)	1.16 ± 0.14	0.54 ± 0.14	1.55 ± 0.28	3.38 ± 0.94
Mean inter-burst instant freq (Hz)	—	0.33 ± 0.03	0.19 ± 0.03	0.27 ± 0.07
Mean intra-burst instant freq (Hz)	—	123.7 ± 4.3	15.1 ± 2.5	33.5 ± 4.6
	**RS**	**RFB**	**RSB**	
No. Neurons	49	4	22	
% total	13.2%	1.1%	5.9%	
Mean frequency in 5 min (Hz)	5.62 ± 0.38	7.40 ± 4.68	4.35 ± 1.08	
Mean inter-burst instant freq (Hz)	—	4.11 ± 2.48	0.31 ± 0.07	
Mean intra-burst instant freq (Hz)	—	137.8 ± 8.8	13.1 ± 1.3	
CV for classification	0.28 ± 0.02	0.32 ± 0.05	0.39 ± 0.02	
CV intraburst	—	0.16 ± 0.05	0.9 ± 0.1	

Approximately 80% of neurons recorded showed irregular firing patterns of spontaneous activity. Within this group of neurons, more than half showed single spikes and were classed as Irregular Simple neurons (or IS) whereas the remaining had >25% of their spikes grouped in bursts. Bursting neurons were further subdivided into three classes according to the traits of their bursts. Irregular Fast Burst neurons (or IFB) produced shorts bursts (mean duration 39 ± 5 ms) of high frequency intra-burst spiking (see Table 1). Neurons of this class fired a mean of 2.9 ± 0.1 spikes. Irregular Slow Burst neurons (or ISB) showed longer bursts (mean duration 8.2 ± 2.4 s) with many spikes (27.6 ± 6.8) and low intra- and inter-burst spike frequency (see Table 1). A third class of neurons showed bursts (mean duration 2.6 ± 0.9 s; mean number of spikes 39.6 ± 18.9) with variable frequency, typically high at the starting and low at end of burst. These were termed Irregular Mixed Burst neurons (or IMB).

A 20% of neurons in the present sample exhibited regular firing patterns. A neuron was classed as regular when the CV of their spike intervals was under 0.5^14^. Regular Simple neurons (RS) produced single spikes at regular intervals. We had previously labelled these as clock-like^5^. Using the CV method, here we report additional regular patterns. Regular Slow Burst neurons (RSB) produced bursts of spikes at regular intervals such that the CV applied only to the first spike of each burst was < 0.5 (see Table 1). Burst properties of these neurons were like those of irregular neurons (mean number of spikes was 33 ± 9; mean duration 5.7 ± 1.6 s) and intra-burst spiking was regular or irregular (see Table 1). A few Regular Fast Burst neurons (RFB) were also found (mean number of spikes was 2.3 ± 0.2 and mean duration 10 ± 2 ms). This latter class of neurons typically showed double regularity inter- and intra-burst (see Table 1). No mixed bursting neurons were found to follow any identifiable regularity.

**Figure 1:**
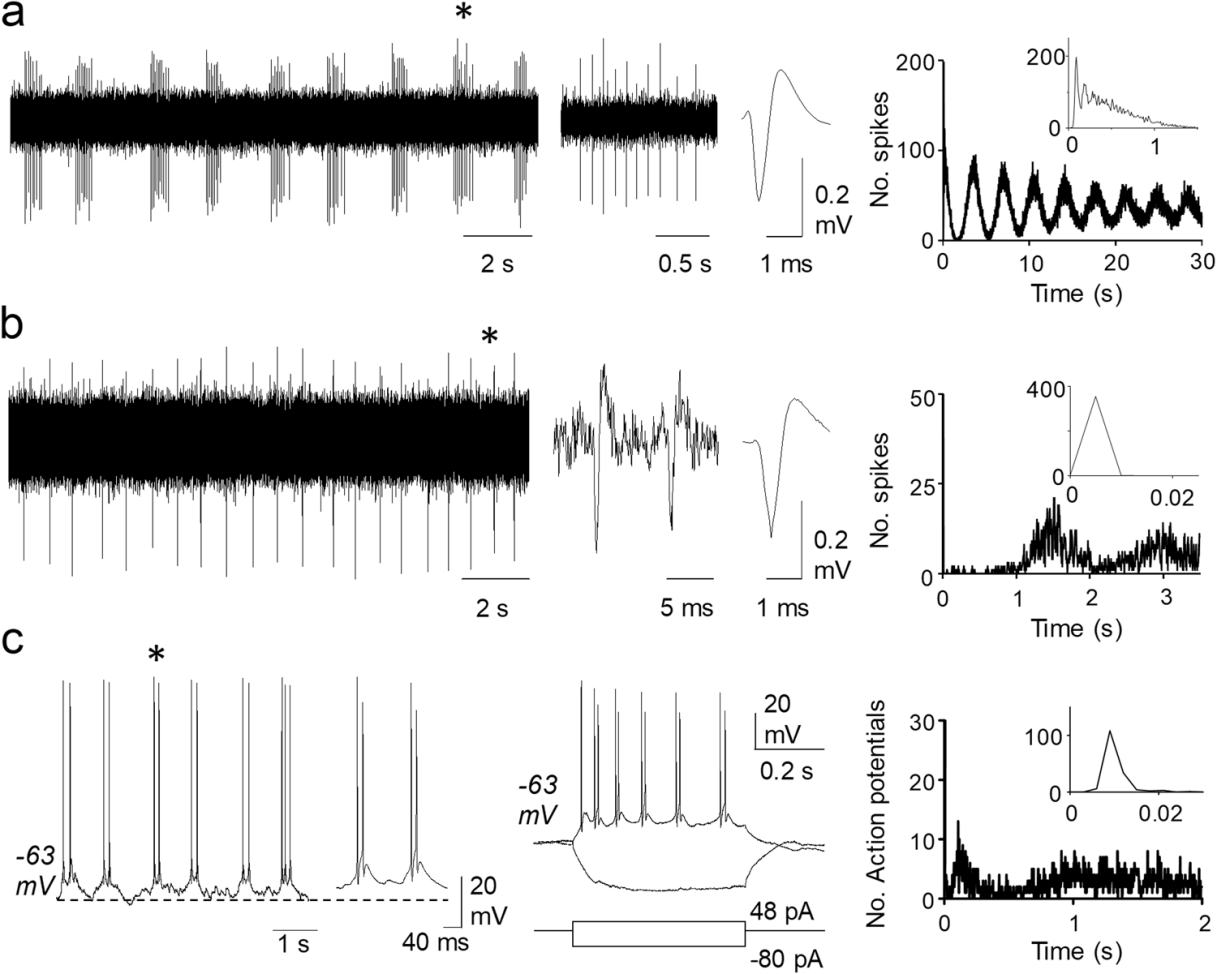
Regular bursting patterns. (**a)** Shows an original record of a slow burst pattern followed by an expanded time base record of one burst (marked by an asterisk in the original) and an averaged spike. The autocorrelogram at the right-hand side shows inter-burst regularity and the inset shows a peak indicative of intra-burst regularity. (**b)** Shows original recordings of a fast burst neuron and the same sequence of images as in a. The neuron fired duplets at a high intra-burst frequency. (**c)** Shows voltage recordings from the only regular fast burst neuron which was recorded in whole cell mode. This neuron fired bursts of two duplets as shown in the expanded time base record. In the middle panel, responses of this neuron to current pulses showing an unusual response to depolarisation, firing spike duplets. The autocorrelogram is like that of neuron in b. All three neurons passed regularity tests and had inter-burst CV values < 0.5.

Examples of regular bursting neurons not previously reported by our group are shown in Fig. 1.

### Effects of synaptic blockade on spontaneous activity

**Figure 2:**
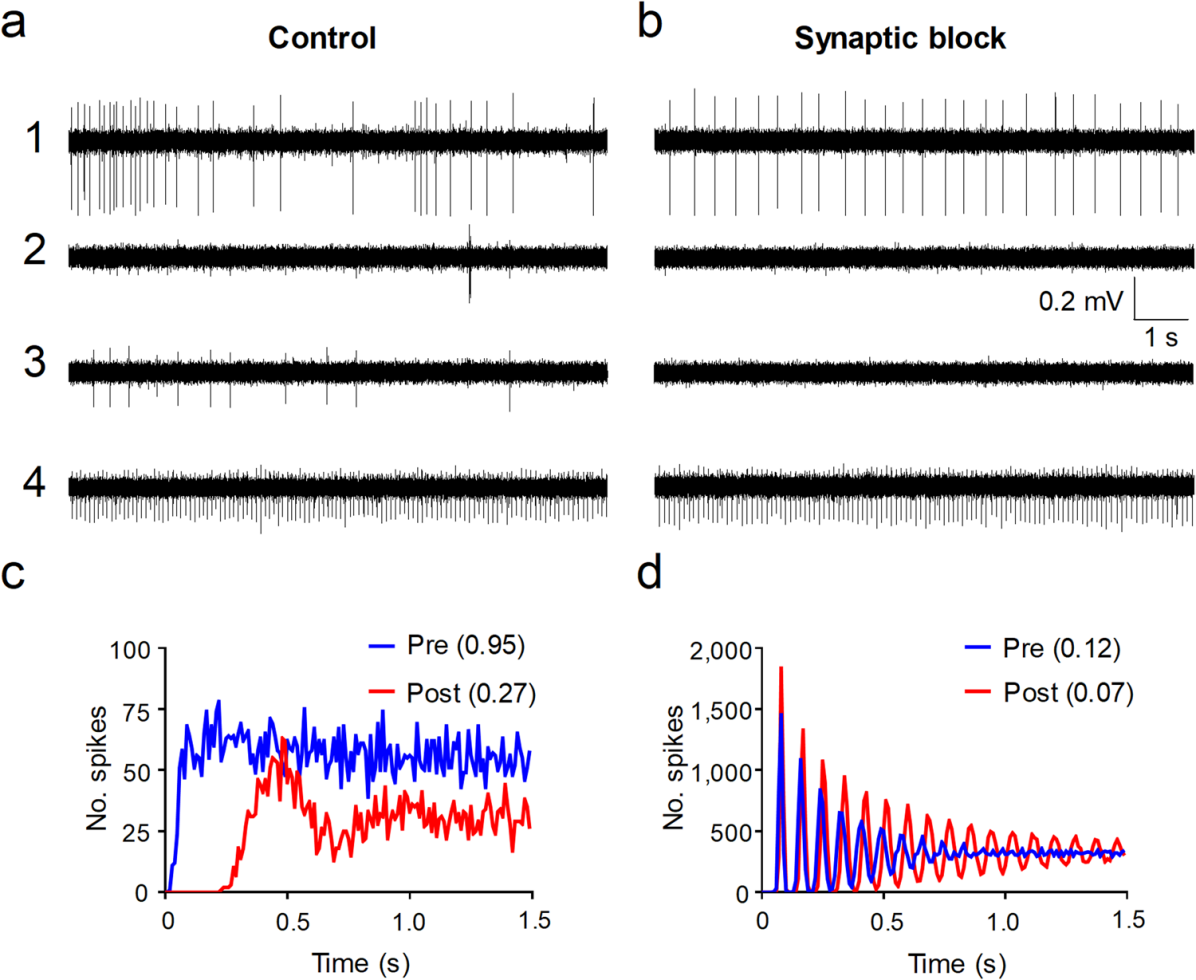
Effects of synaptic blockade on spontaneous activity. (**a)** Shows an original extracellular synchronous recording from 4 separate recording channels under control conditions. Recordings in 1 correspond to an irregular simple pattern, in 2 to an irregular fast burst, in 3 to another irregular simple and that in 4 to a regular simple pattern. (**b)** Shows simultaneous recordings obtained from the same neurons after combined superfusion of synaptic blockers. Calibration bars apply to a and b. The neuron in 1 became regular as shown in the autocorrelograms in (**c)**. Neurons in 2 and 3 were inhibited. The neuron in 4 increased its regularity as shown in autocorrelogram (**d)**. Coefficients of variation (CV) before (Pre-) and after (Post-) synaptic blockade are shown in brackets.

Two different cocktails of synaptic inhibitors were used. The simple cocktail contained only NBQX (5 µM) and picrotoxin (PTX; 20 µM) to block the main excitatory and inhibitory amino acidergic synapsis. The complete cocktail included, in addition, D-AP5 (50 µM), strychnine (5 µM) and CP9994 (10 µM) to complete the blockade of amino acid- as well as peptidergic transmission. Cocktails were superfused for 30 min while spontaneous activity was monitored. Figure 2 shows a representative experiment of this series showing spike trains inhibited, modified and insensitive to synaptic blockade.

Contingency analysis showed that there were no differences between the use of simple or complete cocktails regarding their inhibitory effects on spontaneous activity in our sample of neurons and therefore datasets were pooled together for analysis. The effects of synaptic blockade were tested in 153 neurons of which 118 fired irregularly and the remaining 35 showed regular firing. Synaptic blockade produced regularisation in the firing of 10 neurons that were reclassified according to the post blockade pattern. In addition, 98 out of 108 irregular neurons had their spontaneous activity inhibited whereas only 8 out of 45 regular neurons were blocked. Table 2 shows these results distributed by neuronal class. Contingency analysis shows that irregular neurons were inhibited by the synaptic blockers more frequently than regular neurons (Chi-square, p < 0.0001). In contrast, we cannot conclude that any subtype of irregular neuron is more sensitive to synaptic block than others.

**Table 2:** Effect of synaptic blockers on the different firing patterns of spontaneous activity.

	Simple	Slow Burst	Fast Burst	Mixed Burst	Totals
Irregular	61/68 (89.7%)	4/4 (100%)	31/33 (93.9%)	2/3 (66.6%)	98/108 (90.7%)
Regular	7/34 (20.6%)	0/9 (0%)	1/2 (50%)	—	8/45 (17.7%)

Inhibited/total (percentage) of neurons per neuronal class after application of cocktails of synaptic blockers (pooled data).

The few regular neurons which were sensitive to synaptic block belonged to the RS class (n = 7) and to the RFB (n = 1). These RS neurons had a mean frequency of 3.8 ± 0.4 Hz (lower than the mean of their group, p < 0.05). Irregular neurons insensitive to synaptic block belonged to the IS (n = 7), IFB (n = 2) or IM class (n = 1). No obvious differences were found between these neurons and the majority of irregular neurons that were sensitive to synaptic block.

Neurons that became regular after synaptic block belonged to the IS class (n = 7) or the ISB class (n = 3). The latter had CVs close to the threshold for inclusion in the regular class and synaptic block introduced only a slight change in their spontaneous pattern. One of these neurons acquired a double regularity (ie.- inter-burst and intra-burst). In contrast IS neurons that became RS, had large CVs before synaptic block (mean CV was 1.6 ± 0.4) and changes in pattern were obvious (mean CV after synaptic block was 0.3 ± 0.04; see Fig. 2). Although the mean firing frequency of this latter group did no change, some neurons increased and other decreased their firing frequency after synaptic block. The mean CV of regular neurons decreased slightly after synaptic blockade although the group difference was not significant (0.27 vs 0.24; p = 0.16).

### Effects of ion channel blockers on superficial dorsal horn neurons

A series of patch clamp experiments were performed to obtain data on the intrinsic properties of spontaneously active neurons, on the presence of I_h_ and I_NaP_ currents in superficial dorsal horn neurons and to validate the channel blockers used.

Although excitatory and/or inhibitory subthreshold activity was present in all neurons, only 28 out of 150 neurons recorded showed spontaneous action potential firing. Of these only 3 neurons had regular firing. A comparison of electrophysiological traits between neurons with spontaneous firing vs subthreshold activity is shown as supplementary material (Supplementary Table 1).

Spontaneously firing neurons had a more depolarised resting potential, a higher capacity, a more negative action potential threshold and a narrower action potential than neurons with subthreshold activity. Intracellular current injection revealed tonic firing, initial burst and single spike patterns in spontaneously firing and subthreshold neurons, but the delayed firing type was only found in subthreshold neurons (see Supplementary Table 1). A rare observation shown in Fig. 1 depicts a RFB neuron recorded intracellularly which showed a peculiar firing pattern to depolarising current pulses consisting in the firing of duplets at regular intervals.

**Figure 3:**
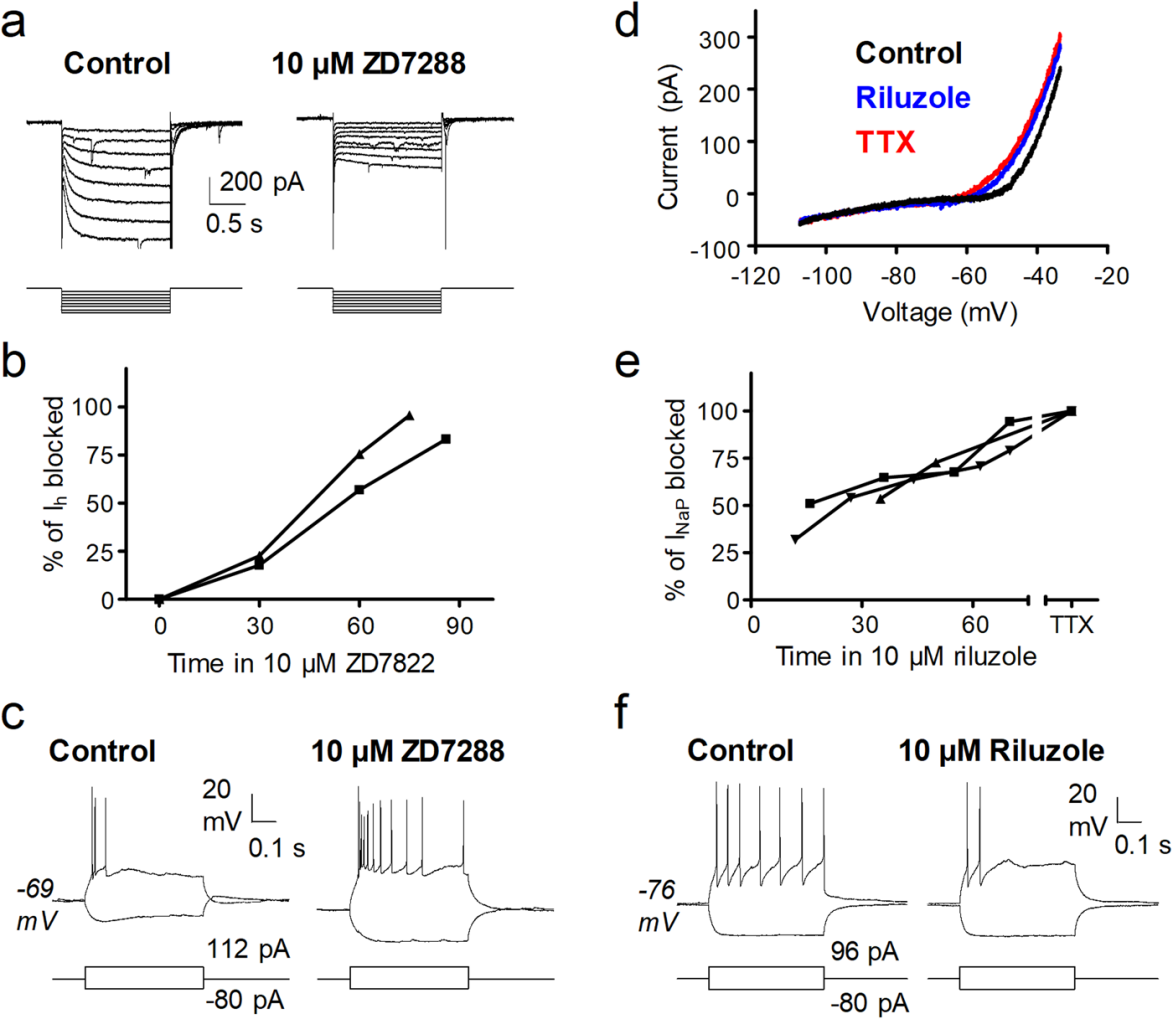
Effects of ZD7288 and riluzole on I_h_ and I_NaP_. The I_h_ was activated using negative voltage pulses (**a**, left). After the application of 10 μM ZD7288 for 86 minutes (**a**, right) most of the current was blocked. Graph in (**b)** shows the slow time course of I_h_ block in the 2 neurons in which a long-lasting application of ZD7288 was successful (neuron shown in A is represented with squares). Voltage recordings in (**c)** show the effects of I_h_ blockade on intrinsic excitability as obtained from the same neuron depicted in a. In this neuron the application of ZD7288 for 72 minutes increased action potential firing in response to depolarising current pulses, abolished sag, increased input resistance and induced membrane potential hyperpolarisation. Graph in (**d)** shows the current recorded in response to a voltage ramp from −107 to −33 mV (26 mV/s) in control conditions (black), after the perfusion of 10 μM riluzole for 70 min (blue) and after the further addition of 0.5 µM TTX for 30 min (red). The results obtained in the 3 neurons in which TTX was applied after riluzole are summarised in (**e)** (neuron shown in d is represented by upside down triangles). The graph was constructed considering that TTX produced a full block (100%) of the I_NaP_. Note that even a long-lasting application of riluzole was unable to achieve a full block of the current. Recordings in (**f)** show a representative example on the depressant effects of riluzole (applied for 60 minutes) on intrinsic excitability of superficial dorsal horn neurons (the time course of I_NaP_ block in this neuron is represented by the squares in e).

Hyperpolarisation-activated currents were detected in spontaneously firing and subthreshold neurons with equal proportions; however, firing neurons had a significantly larger current density (see Supplementary Table 1). The I_h_ as well as the associated sag observed in current clamp records were blocked by ZD7288 applied at 10 µM in 2 out of 2 neurons tested. It took very prolonged periods of drug superfusion to produce a near complete blockade. During superfusion of ZD7288, both neurons hyperpolarised slightly and increased their membrane resistance (see Fig. 3).

The presence of I_NaP_ in the recorded neurons was inferred by the existence of a negative inflexion at potentials positive to −70 mV in the current response to depolarising voltage ramps. This inward current was detected more frequently in spontaneously firing neurons than in subthreshold neurons (see Supplementary Table 1). Confirmation of the nature of the current was obtained in 11 neurons using two blockers of the persistent sodium currents, riluzole (10 µM) and tetrodotoxin (TTX, 0.5 µM). In 3 neurons TTX was applied during 30 min after perfusion of riluzole (Fig. 3). In these experiments riluzole showed a slow and incomplete blockade of the current (to 82.1 ± 6.4% of reduction). Estimates of current density (0.78 ± 0.20 pA/pF; n = 6) and voltage for half maximal activation (−51.7 ± 1.3 mV; n = 3) were obtained from experiments in which TTX produced a full block of the current.

### Effects of ion channel blockers on spontaneous activity

**Figure 4:**
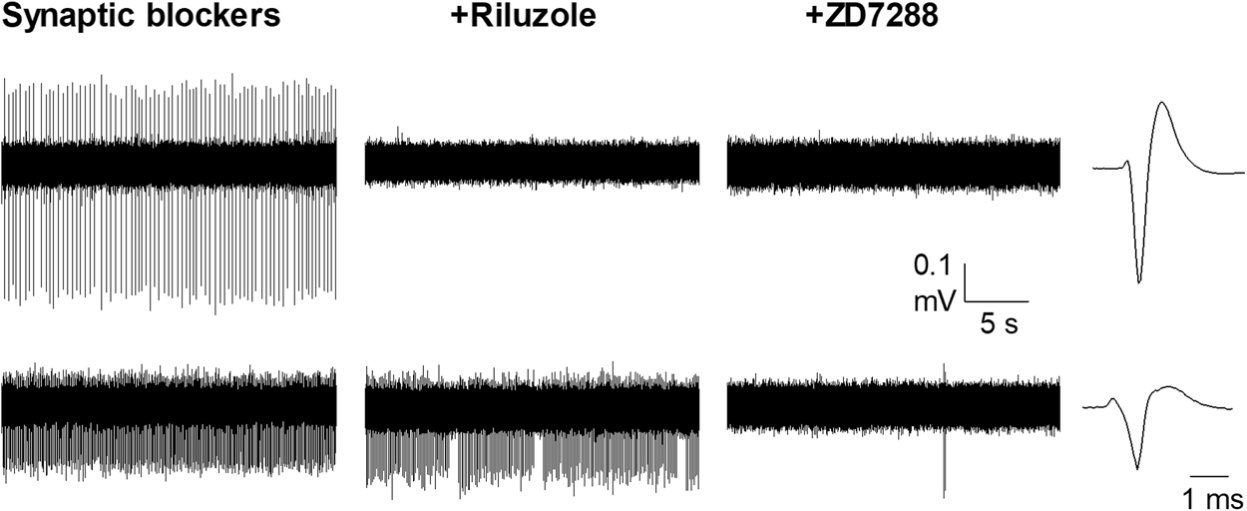
Effects of ion channel blockers after synaptic blockade. Figure shows simultaneous recordings from 2 different regular simple neurons resistant to synaptic block (same as shown in Fig. 2a1 and a4). Neurons were recorded in ACSF containing synaptic blockers (left panel), 45 min after addition of riluzole (10 µM, middle pannel) and 90 min after addition of ZD7288 (10 µM, right panel) to the superfusate as labelled. Calibration bars apply to all three conditions. Spike averages for each neuron are shown in an expanded time base (far right).

Following synaptic blockade, ion channel blockers were added to the ACSF and their effects on the spontaneous activity were assessed. Figure 4 shows a representative experiment and Table 3 presents detailed data on drug inhibition per neuronal class. The effects of ZD7288 were studied in a total of 27 neurons whose spontaneous activity was resistant to synaptic blockade. In agreement with the previous patch clamp studies ZD7288 was superfused for prolonged times (90 min). This compound blocked spontaneous firing in 20/27 neurons (see Table 3).

**Table 3:** Effects of ion channel blockers on firing patterns sensitive and resistant to synaptic block.

	After Synaptic Blockade			Without Synaptic Blockade		
	ZD7288	Riluzole	Riluzole + ZD	ZD7288	Riluzole	Riluzole + ZD
IS	2/3 (66%)	3/3 (100%)	3/3 (100%)	34/41 (83%)	21/31 (68%)	23/24 (96%)
ISB	—	—	—	8/10 (80%)	4/4 (100%)	3/3 (100%)
IFB	—	—	—	7/8 (87%)	14/18 (78%)	6/7 (86%)
Mixed	1/1 (100%)	0/1 (0%)	1/1 (100%)	1/1 (100%)	0/4 (0%)	—
All Irregular	—	—	—	**50/60 (83%)**	**39/57 (68%)**	**32/34 (94%)**
RS	13/18 (72%)	6/12 (50%)	11/12 (91%)	3/3 (100)	3/6 (50%)	4/4 100%)
RSB	4/5 (80%)	4/8 (50%)	7/8 (87%)	2/2 (100%)	0/3 (0%)	2/2 (100%)
RFB	—	—	—	—	1/2 (50%)	1/1 (100%)
All Regular	**17/23 (73%)**	**10/20 (50%)**	**18/20 (90)**	5/5 (100%)	4/11(36%)	7/7 (100%)
Totals	20/27 (74%)	13/24 (54%)	22/24 (91%)	55/65 (84%)	43/68 (63%)	39/41 (95%)

Number of inhibited/group total neurons after application of ZD7288, riluzole or a combination of both. Data on riluzole and riluzole + ZD columns obtained sequentially from some experiments.

Riluzole was applied to 24 neurons with spontaneous activity resistant to synaptic blockade and this activity was inhibited in 13 neurons (see Table 3). In this series of experiments, ZD7288 was added to the superfusate to test the combined effects of riluzole and ZD7288 (see Table 3). After combined application of both ion channel blockers only 2/24 neurons retained their spontaneous activity.

In a different experiment, ion channel blockers were applied in the absence of synaptic blockers to test whether they could modulate spontaneous activity in irregular neurons as well (see Table 3). Results show that ZD7288, riluzole and riluzole + ZD7288 produced inhibition of irregular patterns of spontaneous activity in a large majority of neurons.

### Effects of ion channel blockers on segmental transmission

Finally, we applied the ion channel blockers to whole cord preparations while recording ventral root responses to dorsal root stimuli. Fifteen C-fiber intensity electrical stimuli were delivered to evoke a windup response in a population of motor neurons. This experiment was performed using mice pups (7 day-old) since entire cords from adult animals survive poorly under *in vitro* conditions. Representative recordings of this experiment as well as quantitative analysis of results are presented in supplementary materials (Supplementary Figure 1).

Cumulative applications of riluzole at 1, 3 and 10 µM (n = 4) produced a concentration dependent inhibition on windup and the underlying cumulative depolarisation. Drug-induced inhibition of spike firing was visible at 1 µM and almost complete at 10 µM. Cumulative depolarisation was reduced only by 3 and 10 µM applications of riluzole.

As previously reported^15^, ZD7288 had the opposite effect on segmental transmission. Under the present conditions, application of this compound at 1, 3 and 10 µM (n = 4) had a consistent and strong concentration-dependent excitatory effect on spike firing and the underlying cumulative depolarisation.

## Discussion

Using multielectrodes and a flexible semi-automated application for the analysis of spike trains we report the presence of a variety of spontaneous firing patterns among superficial dorsal horn neurons. This classification includes four types of neurons with irregular spiking and another three types of neurons with regular or rhythmic spiking behaviour. Two types of irregular bursting neurons (ISB and IMB) have not been described before by other groups although they have been described in a previous report from our group^5^. As explained in detail in the introduction all previous reports focus on one or several patterns but do not mention the wide array of types presented here.

One major contribution of our present work is to establish a comprehensive classification of superficial dorsal horn neurons according to their spontaneous activity. It is possible that a wider sample of neurons leads to the discovery of new classes of neurons not included as such in our proposal. In this line, we report some bursting neurons with a double regularity (inter- and intra-burst) but the number of observations is too low to create a separate category. Some authors have described neurons firing high frequency spike duplets^16^ which we have included in the fast burst class. In our hands many fast bursting neurons that fire duplets, occasionally may produce bursts with 3 o 4 spikes at high frequency. Taken the framework of our present classification, we have tried to unify criteria and establish the equivalence with previously reported patterns which is not a straight forward issue due to the succinct and in occasions incomplete descriptions offered in the written reports. This is shown in Table 4 which we hope will be a helpful reference for future work.

**Table 4:** Summary of previous publications on spontaneous activity of dorsal horn neurons.

	Cervero *et al*.^7^	Sandkühler & Eblen-Zajjur^3^	Rojas-Piloni *et al*.^16^	Li & Baccei^4^	Luz *et al*.^11^	Zhang *et al*.^19^
IS	Inverted (inhibitory)	Non-rhythmic		Irregular	Rhythmic	
IFB		Non-rhythmic^a^	Duplets^a^			
ISB						
IMB						
RS		Rhythmic		Tonic	Rhythmic	GABA-ergic
RFB		Rhythmic^a^				
RSB				Bursting^b^		
LAMINA	L II	L I-VI	L I	L I	L I	L II
PREPARATION	Acute cat	Acute rat	Acute rat	Slice rat	Slice rat	Slice mice

^a^Bursts are not clearly defined but analysis shown are consistent with our fast burst type.

^b^Bursts are not clearly characterised, examples shown are consistent with our slow burst type.

References^4,11,19^ do not include a rigorous statistical analysis of regularity.

A direct observation of the literature as well as the results presented here, shows that neurons firing single spikes and bursts of spikes with or without regularity have been reported under different experimental conditions *in vivo* and *in vitro* by several groups. This indicates that spontaneous activity is not an artefact but rather a phenomenon that has an explanation and, most likely, a physiological function.

The identity of neurons recorded has not been established in the present report. Due to the preparation used and the location of electrodes in superficial laminae we assume that a high percentage of neurons of our sample are local interneurons based on previous studies^17,18^. It has been shown that a considerable number of GABAergic neurons possess spontaneous activity probably of the regular simple type^19^. We believe that they form a network of interconnected neurons performing operations related to somatosensory processing, particularly involved in nociception. Recently we have shown that a peripheral nerve lesion increases the numbers of neurons with a regular simple pattern and the synchrony of irregular fast burst neurons^5^. In addition, spontaneous activity can be altered by TNF alpha, a proinflammatory cytokine^19^. However, it has been shown that projection neurons in both dorsal and ventral pathways display spontaneous activity. A recent study^10^ convincingly demonstrates that lamina I spino-mesencephalic projecting neurons show spontaneous activity probably of the regular fast burst type.

A second valuable contribution of the present work is the establishment of some clear-cut ideas about the genesis of spontaneous activity in the superficial dorsal horn. The generation of spontaneous events in neuronal networks may depend on the integration of a variety of factors including synaptic input and endogenous rhythmogenesis. The present experiments show that synaptic blockade stops spontaneous firing in most irregular neurons regardless of the specific pattern presented by the neuron. The very few irregular neurons that did stand synaptic blockade (<10% in our sample) could have been driven by neurotransmitters not fully blocked by our inhibitors. Endogenously generated activity comes often associated to regular firing; however, it is possible to envisage complex ionic mechanisms capable to produce irregular activity. In agreement with previous work on dorsal horn neurons, we also report that regular patterns are insensitive to synaptic blockade^4,11^. The few regular neurons sensitive to synaptic blockade may have been driven by neurons with endogenous activity via strong mono-synaptic connections. In fact, synaptic blockade appears to favour rhythmicity unveiling an underlying regularity in some neurons and decreasing the value of the coefficient of variation in others. These observations altogether suggest that endogenously generated rhythmic activity is at the origin of all spontaneous activity observed in the superficial dorsal horn.

The mechanisms that generate endogenous rhythmic events (single spikes or bursts) are varied and complex. Recent studies suggest that there are several conductances that are basic to generate rhythmicity and others that may be involved in the modulation of intervals between spikes or spikes per burst^20,21^. Other mechanisms such as the regulation of the extracellular concentration of ions by astrocytes or transient intracellular calcium waves are currently discussed in neurons and heart pacemakers^21,22^. Most researchers agree in that hyperpolarisation activated cationic currents and persistent sodium currents are among the principal conductances necessary to produce endogenous rhythmicity.

The I_h_ has been shown to be involved in the generation of rhythmicity in heart pacemakers^22^, in thalamic neurons^23,24^ and neurons of the suprachiasmatic nucleus^25^. In agreement with previous reports^15^, our present results suggest that the I_h_ is expressed in many dorsal horn neurons although the current density is significantly larger in neurons that fire spontaneous action potentials. The current may be particularly large in a subset of spinal neurons with regular activity^26^. The present experiments show that ZD7288 applied at 10 µM during a prolonged time produces an almost complete blockade of the current recorded in voltage clamp mode as well as the sag and rebound depolarisation seen during hyperpolarising current pulses in current clamp recordings. Under these conditions, ZD7288 produced the blockade of rhythmic activity in neurons with single and bursting behaviour. Up to a 70% of neurons with rhythmic patterns stopped firing during prolonged perfusion of ZD7288. Our data suggest that none of the specific patterns of regular neurons is particularly sensitive or insensitive to I_h_ blockade confirming the fundamental role of this current in the generation of rhythmicity. The I_h_ may work as a regenerating current activated when the membrane potential reaches the lower values after an event to depolarise it and generate a new upstroke leading to the next event. In conjunction with other low threshold calcium currents this mechanism may produce rhythmic bursting behaviour. The blockade of the current may prevent the generation of cycles and, in addition, it may hyperpolarise neurons decreasing the probability of firing.

The I_NaP_ has been shown to be involved in rhythmogenesis of many neurons including respiratory motorneurons^27^, trigeminal neurons involved in the control of mastication^28^, supragranular cortical neurons^29^ or hippocampal pyramidal neurons^30^. This current is known to be present in dorsal horn neurons^31,32^ and regular slow burst neurons appear to have a characteristic high ratio of persistent sodium current to leak currents^4^. The latter authors showed that riluzole can produce a reduction of bursting behaviour in these neurons. Using whole cell recordings, we report here that persistent sodium currents are more often found in neurons with spontaneous suprathreshold activity that in those with subthreshold activity. Unfortunately, the present sample of neurons recorded with whole cell techniques did not allow us to obtain data indicating whether rhythmic neurons have stronger currents than other neurons. In our hands riluzole applied for a prolonged period produces a strong blockade of the current (up to 80%). Using this pharmacological tool, we show here that riluzole can block regular activity in single as well as bursting neurons; however, not all regular neurons respond to riluzole indicating that persistent sodium currents are necessary to produce rhythmicity only in a subset of neurons, approximately 50%. The I_NaP_ is an auto-regenerating depolarising current important at the upstroke of the cycle. This current is capable to produce bursting behaviour in the absence of calcium^33^ and therefore, the resulting pattern induced by this current may depend on the presence of repolarising potassium or chloride currents.

The percentage of rhythmic neurons sensitive to a mixture of riluzole and ZD7288 increases to 90%. This suggests that some rhythmic neurons may express either of the currents, some others may express both and a few more express neither. From this observation we can conclude that both the I_h_ and the I_NaP_ are necessary to produce rhythmicity in large proportions of superficial dorsal horn neurons, but it seems clear that other currents may be involved. Studies of spiking rhythmicity suggest that calcium-activated non-selective cationic currents as well as a variety of outward potassium currents play fundamental roles in the production of cycles.

It is also of great interest the observation that ion channel blockers produce a strong inhibition of irregular spontaneous activity affecting 94% of neurons. It is very likely that this is due to the blockade of rhythmic activity that may be the largest source of synaptic drive for irregular neurons. However, since the currents are expressed in many dorsal horn neurons, a direct post-synaptic effect of the channel blockers on irregular neurons to limit their synaptic responses cannot be excluded.

The wide expression of I_h_ and I_NaP_ in the spinal cord precludes the use of the channel blockers as appropriate tools to investigate the physiological function of spontaneous activity in the dorsal horn. HCN and sodium channels are present in dorsal and ventral horn neurons as well, many of which do not show spontaneous activity^34,35^. Our dorsal root-ventral root experiments illustrate this point. Whereas both I_h_ and I_NaP_ blockers produce a strong reduction of the spontaneous activity in the dorsal horn, they have clear-cut opposite effects in the modulation of reflex pathways. It would be possible to explain these effects on the speculation that neurons inhibited by each of the blockers are involved in different processes leading to excitation and inhibition of reflexes. However, as explained before^15^, application of ZD7288 and the consequent increase in membrane resistance of neurons involved in the processing of reflex responses at dorsal and ventral areas may potentiate transmission. In contrast, blockade of I_NaP_ has an inhibitory effect decreasing the probability of firing. Therefore, the results of these experiments are more plausibly explained by actions of the channel blockers in many spinal neurons and not just in those with spontaneous activity.

It will be necessary to understand better the biophysics of rhythmicity and find specific tools capable to block single patterns of activity that eventually may reveal the function of these neurons. So far, the physiological function of rhythmic firing in the dorsal horn has been understood in terms of a possible role in the development of the nociceptive system^36^ but also as a mechanism for the coding of nociceptive information^5^. The coding role of these neurons is so far unexplored, but many possibilities can be envisaged. Somatosensory stimuli are converted into trains of action potentials by peripheral receptors so that primary afferents use a rate-code strategy to convey information into the spinal cord. However, the spinal output code is likely to use a temporal code in which regular activity may serve as an internal clock that enables precise temporal detection of stimuli in brain structures^37^. Further research is required to establish the role of these neurons.

## Methods

### Animals

Experiments were performed in a total of 109 CD1 female mice (31–57 day-old) and 8 neonatal mice of both sexes (6–10 day-old). Experiments were designed following European Union and Spanish Government regulations and the experimental protocols were approved by the University of Alcala Ethics Committee and the Govern of the Community of Madrid (PROEX 018/16).

### Preparation of spinal cord slices

The procedure for preparation of the slice was described previously^5^. Briefly, thoracic to sacral regions of the cords were extracted under deep anaesthesia (urethane 2 g/Kg i.p.) and placed in cold sucrose-substituted artificial cerebro-spinal fluid (ACSF) to remove meninges and cut dorsal roots except L3, L4 and L5. Then a single horizontal slice of 500 µm containing the dorsal horn and dorsal roots was obtained in a vibratome. The slice was pinned down to soft based recording chamber, with its ventral surface upwards, and maintained at 22 ± 1 °C with oxygenated ACSF at a flow of 5 ± 1 mL/min. The composition of ACSF was (in mM) NaCl 127, KCl 1.9, KH_2_PO_4_ 1.5, MgSO_4_ 1.3, CaCl_2_ 2, NaHCO_3_ 22, and glucose 10, (pH 7.4). In the sucrose-substituted ACSF, NaCl was substituted with an equimolar concentration of sucrose.

### Extracellular recordings with MEAs

Extracellular action potentials were recorded using multielectrode arrays consisting of 16 recording sites (MEAs, Neuronexus Technologies, USA) following a procedure previously described^5^. Approximately one half of the neurons sampled were recorded within a new recording set up. A 32-recording site electrode from Neuronexus Technologies was used (Buzsaki 32-A32, 4 shanks with 8 recording sites each). The electrode was connected to a RHS2000 32- channel stimulation/recording headstage containing two RHS2116 amplifier chips (Intan Technologies, USA), the signal was band-pass filtered between 200 Hz and 3 KHz, amplified and digitalised at 20 KHz. Signals were transferred to the computer, converted into Spike 2 compatible format and stored for offline analysis using Spike 2 (CED, UK).

Electrodes were placed over the slice using a micromanipulator. The electrode was rapidly lowered into the slice until its tip reached the ventral limit of laminae II (~300 µm) and then moved down slowly at 10 µm steps until finding an appropriate recording position in which the spontaneous activity of several neurons could be distinguished.

### Spike sorting and spike-train analysis

Spike sorting was performed with Spike2 sorting software using principal component analysis, based on spike shape parameters and multichannel analysis. Firing patterns were defined according to regularity, grouping of spikes in bursts and firing frequency inter- and intra-bursts. Neurons were classified as burst firing if at least 25% of their spikes appeared grouped. The criteria to distinguish fast from slow bursts was the mean intra-burst spike frequency, being 70 Hz the limit between groups. Regularity was measured using an in-house developed application (in Matlab) which allowed a semi-automatic quantification of the coefficient of variation (CV) of inter-spike intervals (ISI). This application allowed to measure ISI as well as inter-burst intervals (IBI) and to measure the CV considering all intervals, ISI or IBI. The application has been made available to the public in an open code website (link: https://github.com/Lucas-Romero-J/App_Spike_Train_Analysis)

Mean firing frequency was measured 5 minutes prior to administration of any drug and during the last 5 min of perfusion of each compound. Drugs were administered for 30–90 min periods. The spontaneous activity of a neuron was considered inhibited if its firing frequency was reduced to ≤20% of control.

### Dorsal root-ventral root recordings

Dorsal root-ventral root recordings were performed in whole spinal cord preparations obtained from 6–10 day-old mice following a routine previously reported^38^. Briefly, following cord extraction under anaesthesia it was placed in a recording chamber and maintained under the same conditions that the slice previously explained. The L4 or L5 dorsal root and its corresponding ventral root were placed in tight fitting glass suction electrodes and trains of C-fibre strength stimuli were applied to the dorsal root (15 stimuli at 1 Hz; 200 µs and 200 µA). Ventral root responses were recorded with a DC coupled amplifier, filtered in AC and DC modes, digitalised at 6 KHz and stored for offline analysis. Repetitive stimulation evokes a cumulative depolarisation in the DC channel and an increasing number of action potentials for each stimulus, typical of the wind-up phenomenon. Total spike counts and cumulative depolarisation was measured before and after drug perfusion to evaluate the effects on segmental transmission.

### Whole cell recordings

Electrodes were pulled from borosilicate glass with internal filament (Harvard apparatus ltd, UK) and presented resistances within the range of 5–9 MΩ. An internal solution without calcium chelators was used to enhance the probability of spontaneously firing neurons^4^. This solution contained in mM: 130 K-gluconate, 10 KCl, 10 HEPES, 10 Na-phosphocreatine, Na_2_-ATP 4, Na-GTP 0.3 pH 7.2 (303 mOsm). All procedures were identical to those previously reported^5^. Liquid junction potential (11.5 mV) and series resistances were corrected offline for all recordings.

For current clamp recordings, signals were digitised at 10 kHz and analysed offline with Spike 2 software. Depolarising and hyperpolarising intracellular current pulses of 500 ms were applied to study the firing pattern and the passive electrical properties of neurons like input resistance and cell capacitance^15^. The presence of depolarising voltage sag and rebound depolarisations was also studied^26^.

Voltage-clamp recordings were filtered at 4 KHz, sampled at 10 KHz and stored for offline analysis. Hyperpolarisation-activated currents (I_h_) were activated from a holding potential of −62 mV using 1.5 s duration step pulses between −72 and −132 mV. I_h_ amplitude was measured as the difference between instantaneous and steady-state current, current intensity at −132 mV was divided by cell capacitance to calculate current density^15^.

To study the presence of persistent sodium currents neurons were held at −113 mV for 3 seconds and then a voltage ramp to −31 mV was applied. A ramp rising rate of 26 mV/s was initially used in all neurons^4^, but to avoid action potential firing in control conditions it was adjusted in occasions by lowering rate down to 8 mV/s. Persistent sodium currents were measured by subtracting the recorded current during application of the blockers with that obtained in control conditions. Activation curves were constructed converting current values at each corrected test potential to conductances using the following equation with a calculated ENa of 56 mV: g = I/(V − ENa). Normalised conductances were fitted using a Boltzmann function: g/gmax = Bottom + (Top-Bottom)/(1 + exp((V50 − V)/Slope)) where gmax was the maximum conductance measured, V the corrected test potential and V50 the potential for half activation.

### Drugs and chemicals

The next drugs were used in this work: 1,2,3,4-Tetrahydro-6-nitro-2,3-dioxo-benzo[f]quinoxaline-7-sulfonamide disodium salt hydrate (NBQX), D(−)-2-Amino-5-phosphonopentanoic acid (D-AP5), (2 S,3 S)-3-(2-Methoxybenzylamino)-2-phenylpiperidine dihydrochloride (CP99994), Picrotoxin (PTX), Strychnine, 4-(N-Ethyl-N-phenylamino)-1,2 dimethyl-6-(methylamino) pyrimidinium chloride (ZD7288), 2-Amino-6-(trifluoromethoxy)benzothiazole (riluzole) and Tetrodotoxin (TTX). These drugs and the components of the ACSF and the intracellular solution were purchased from Tocris Bioscience (Bristol, UK) and Sigma Aldrich (Saint Louis, USA). Drugs were dissolved in Milli-Q water or DMSO as concentrated stocks and stored at −20 °C.

### Statistics

All statistical analysis and curve fittings were performed using GraphPad Prism 7.0. Data are presented as mean ± SEM unless otherwise stated. Contingency tables were analysed with the Chi-square test. Comparisons of means were made using parametrical or non-parametric test as appropriate.

## Electronic supplementary material

Supplementary information
