# Supplementary material for: Origin and classification of spontaneous discharges in mouse superficial dorsal horn neurons
Source: Sci Rep. 2018 Jun 27;8:9735. doi: 10.1038/s41598-018-27993-y (PMC6021406; doi:10.1038/s41598-018-27993-y)
Supplement: Supplementary file 1 — Supplementary information [file 41598_2018_27993_MOESM1_ESM.pdf]

Origin and classification of spontaneous discharges in mouse superficial dorsal horn neurons

Javier Lucas-Romero, Ivan Rivera-Arconada, Carolina Roza and Jose A. Lopez-Garcia

Supplementary table 1. Electrophysiological traits of neurons with spontaneous firing and subthreshold activity

	**REGULAR FIRING**	**SPONTANEOUS FIRING**	**SUBTHRESHOLD**
**Number of neurons**	3.0	28	110
**RMP (mV)**	-48.8 ± 2.2	-55.0 ± 1.1*	-62.7 ± 0.6
**Rin (MΩ)**	347 ± 78	539 ± 51	494 ± 23
**Capacity (pF)**	134 ± 31	100.8 ± 8.4*	75.0 ± 3.2
**AP threshold (mV)**	-37.7 ± 0.6	-33.3 ± 0.8*	-27.0 ± 0.5
**AP amplitude (mV)**	88 ± 4	85.9 ± 2.3*	96.5 ± 1.2
**AP width (ms)**	1.0 ± 0.1	1.34 ± 0.07*	1.54 ± 0.04
**Mean rheobase pA (Median value)**	16 (16)	29.7 ± 3.4* (16)	72.2 ± 4.8 (64)
**Rebound firing (Range frequency in Hz)**	2 (140)	16* (13-221)	9 (4-91)
**% of neurons with I_h (No. of neurons with I_h/total)**	100 (2/2)	66.7 (18/27)	61.3 (57/93)
**I_h density (pA/pF)**	-1.28 ± 0.94	-1.31 ± 0.29*	-0.65 ± 0.06
**% of neurons with I_{NaP} (No. of neurons with I_{NaP}/total)**	(1/1)	70 (14/20)*	41.1 (37/90)
**% of neurons I_{NaP} + I_h (I_{NaP}+I_h/total)**	(1/1)	55 (11/20)	34.1 (28/82)
**% of neurons without I_{NaP} nor I_h (No. of neurons/total)**	0.0	10 (2/20)	28 (23/82)
**Firing pattern to injection of depolarising current**	Tonic 3	Tonic 18/52	Tonic 34/52
		Initial burst 8/39	Initial burst 31/39
		Single spike 2/4	Single spike 2/4
			Delayed firing 41/41

Data are shown as mean ± SEM or percentage of total as indicated. Asterisks indicate statistically significant differences between spontaneous firing and subthreshold neurons as obtained using unpaired *t*-test or Chi-square test.

Supplementary figure 1. Effects of ion channel blockers on segmental transmission.

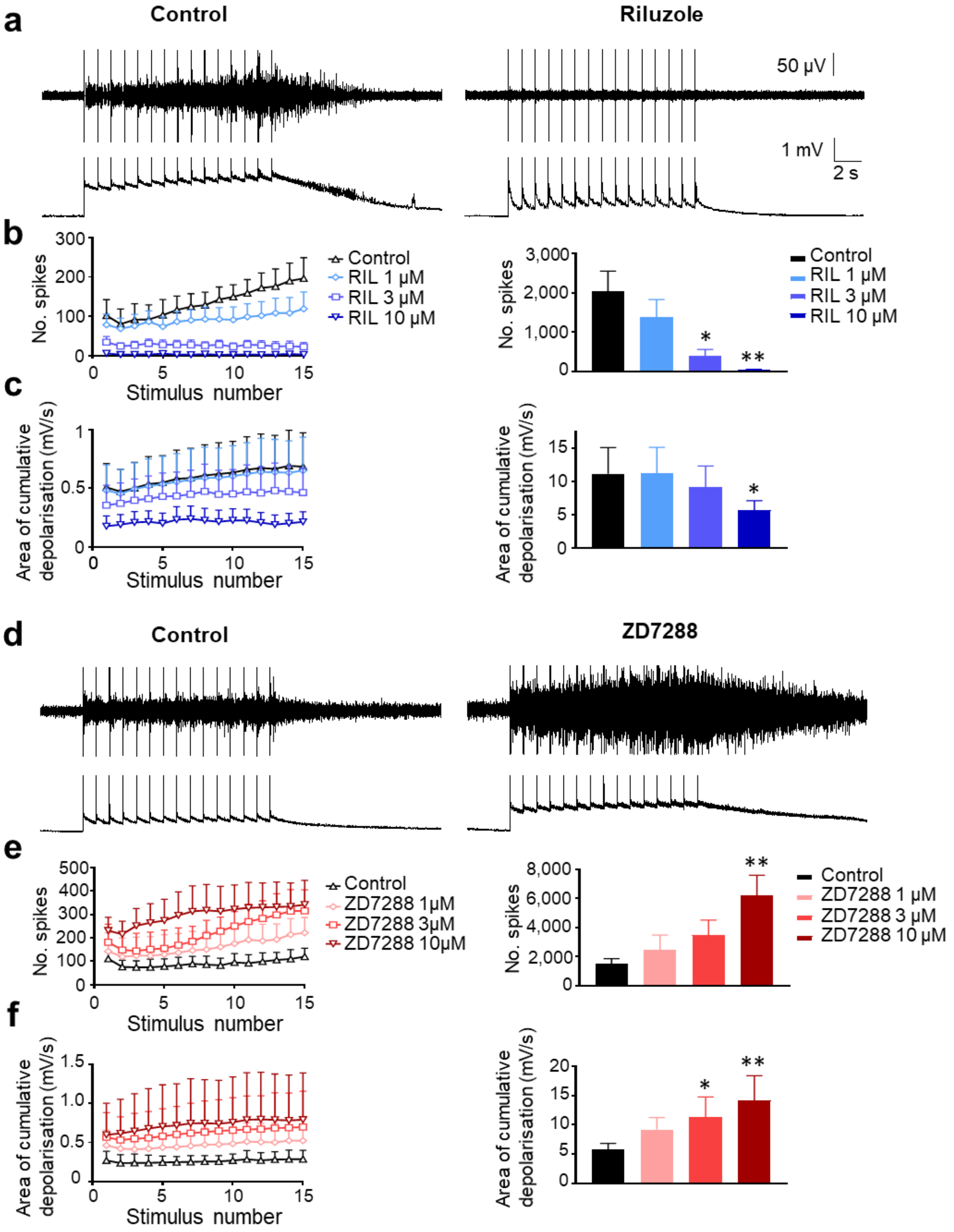

Effects of riluzole and ZD7288 on dorsal root-ventral root wind-up (upper traces) and cumulative depolarisation (lower traces) are shown in **a** and **d** respectively (calibration bars in a apply to d). Graphs show quantitative effects from pooled data. In **b** left, number of events per stimulus in control and in three concentrations of riluzole (cumulative applications) and **b** right total number of spikes per train of stimulus in control and riluzole as labelled. **c** shows effects of riluzole on cumulative depolarisation. **e** and **f** show the same graphs as c and d as obtained during cumulative applications of ZD7288. Further details for methods and analysis can be found in Neuropharmacology, 2016 Oct;109:131-138. doi: 10.1016/j.neuropharm.2016.05.025.
